# P52. A new pathway of tumour antigen loading of human dendritic cells via intercellular communication

**DOI:** 10.1186/2051-1426-2-S2-P26

**Published:** 2014-03-12

**Authors:** G Penna, M Ciranna, F Saccheri, L Morelli, C Pozzi, M Rescigno

**Affiliations:** 1 European Institute of Oncology, Department of Experimental Oncology, Milan, Italy

## Background

Most cancer cells down-regulate gap junctions (GJ) resulting in loss of communication with their surrounding microenvironment. We have previously shown that infection of mouse tumour cell lines with *Salmonella* induces the up-regulation of connexin 43 (Cx43), a ubiquitous protein that forms GJs. This up-regulation allows the transfer of antigens between tumour cells and dendritic cells (DC) licensing them to induce an efficient anti-tumour response in a mouse model of melanoma (Saccheri et al., Sci TM 2010).

Herein, we tested the idea that through the formation of intercellular communication channels in tumour cells, human autologous dendritic cells could be loaded with tumour antigens, preprocessed by cancer cells, and could be used to generate a cancer vaccine.

## Material and methods

Human melanoma cell lines were infected with a vaccine strain of *Salmonella Ty21a* (*Vivotif*) to induce up-regulation of Cx43 and formation of GJ channels. HLA matched DCs were differentiated in vitro by peripheral blood purified monocytes from healthy donors. Expression of surface molecules, cytokine release and cell proliferation was determined by cytofluorimetry. Cytotoxicity was determined either by Delfia cell cytotoxicity assay (PerkinElmer) and by a cytofluorimetry based CD107 assay.

## Results

*Vivotif*-infected tumor cells established GJs in human melanoma cell lines. We show that tumour derived antigens could transit from 'donor' melanoma cells' cytoplasm to DC's cytoplasm through GJs, generate a tumour-specific CTL response and reactivate tumour infiltrating lymphocyte (TIL) in vitro. The transfer of tumor antigens was GJ-dependent because it was abolished in the presence of a GJ-specific inhibitor. In vitro generated tumour-specific human CTLs lysed also non-donor melanoma cell lines indicating that DCs were loaded with tumour-associated antigens shared among melanoma cell lines.

Moreover, in vitro generated CTLs were melanoma specific because they were unable to lyse HLA matched colorectal adenocarcinoma cell lines.

## Conclusions

We exploited an antimicrobial response present in tumour cells to activate cytotoxic CD8 T cells specific for tumour-peptides, through a new pathway of antigen loading of human dendritic cells via intercellular communication channels. This unique and novel approach can be applied across a wide range of tumour cell types and can be used clinically as therapeutic vaccine alone or in combination with gold standard treatments.

